# Supplementary material for: Divalent cations can control a switch-like behavior in heterotypic and homotypic RNA coacervates
Source: Sci Rep. 2019 Aug 21;9:12161. doi: 10.1038/s41598-019-48457-x (PMC6704260; doi:10.1038/s41598-019-48457-x)
Supplement: Supplementary file 1 — Supplementary Information [file 41598_2019_48457_MOESM1_ESM.pdf]

Supplementary Information

Divalent cations can control a switch-like behavior in heterotypic and homotypic RNA coacervates

Paulo L. Onuchic[§a], Anthony N. Milin[§a], Ibraheem Alshareedah[b], Ashok A. Deniz*[a], Priya R. Banerjee*[b]

[a]Department of Integrative Structural and Computational Biology, The Scripps Research Institute, La Jolla, California 92037, USA

[b]Department of Physics, University at Buffalo, State University of New York, Buffalo, NY 14260, USA

§ These authors contributed equally

*Corresponding Authors: P. R. B.: prbanerj@buffalo.edu. A. A. D.: deniz@scripps.edu

Movie Legends

Movie 1: Confocal fluorescence video showing Mg^{2+}-dependent droplet switching. Heterotypic droplets are initially preformed at 10mM Mg^{2+} (0 sec). Addition of 150mM Mg^{2+} results in a window of miscibility (~1 sec). Finally, 500mM Mg^{2+} is added to induce homotypic RNA phase separation (~5 sec) (Conditions: [RP3] = 500µM, 2x polyU wt/wt, 0.5µM RP3-AF594, 0.5µM FAM-UGAAGGAC) (Scale bar = 20µm and speed of movie is 50 frames/second).

Movie 2: Fluorescence video showing reversible dissolution and formation of polyU droplets by consecutive addition of EDTA and $MgCl_2$. We initially start with preformed RNA droplets (0 sec), and upon addition of 50mM EDTA (~2 sec), we observe the disappearance of droplets. Upon addition of 100mM $MgCl_2$ we observe the resurgence of RNA droplets (Conditions: 1.5mg/mL polyU, 35mM $MgCl_2$, 10% PEG, 0.5µM FAM-UGAAGGAC) (Scale bar = 20µm and speed of movie is 50 frames/second).

SI note 1

To model the phase behavior of RP3-poly(U)-Mg^{2+} mixture, we consider the Voorn-Overbeek theory of complex coacervation in the context of Flory-Huggins mean field theory of polymer phase separation[1,2]. For practical purposes, we make a simplified assumption that our system is composed of a polyelectrolyte salt, PR, and an ionic salt, $MgCl_2$. In our model, P is a polycation and Q is a polyanion, having the same size N and the same charge density σ. To ensure electro-neutrality, the two polymers must have equal volume fraction ($\phi_P = \phi_R$). The key idea of this model is to map the complex coacervation, which is an *associative phase separation*, into a *segregative phase separation*. As a starting point, we consider the Voorn-Overbeek free energy of mixing per lattice site, which is given by:

$$\frac{F}{kT} = \frac{\phi}{N}\ln\frac{\phi}{2} + c\ln c + (1-\phi-c)\ln(1-\phi-c) - \alpha(\sigma\phi + c)^{\frac{3}{2}} \quad (1)$$

Where $\phi = \phi_P + \phi_R$ is the volume fraction of the polyelectrolyte salt and c is the ionic salt volume fraction. The effect of salt is considered here by an additional term in the translational entropy as well as the enthalpy part of equation-1. This equation can be mapped into an effective Flory interaction parameter of a polymer-solvent system that phase separates by *segregative mechanism* as described by: [3]

$$\chi_{eff} = \chi + \chi_{ion} = \chi + \frac{2\pi}{3}\frac{l_B^2\kappa^{-1}\sigma^2}{l^3} \quad (2)$$

Where κ^{-1} is the Debye length, l_B is the Bjerrum length and l is the size of the monomeric unit in the polyelectrolyte. The first term represents the short-range excluded volume interaction, which is set to zero (χ=0) for simplicity, as in the case of an athermal solvent[4]. Consequently, the corresponding free energy density equation in the Flory-Huggins framework is given by:

$$\frac{F}{kT} = \frac{\phi}{N}\ln\frac{\phi}{2} + (1-\phi-c)\ln(1-\phi-c) + c\ln c + \chi_{eff}\phi(1-\phi-c) \quad (3)$$

In equation-3, the entropic contribution of salt can be considerable since the salt concentrations used in the experiment are relatively high. The Debye length can be expressed in terms of the ionic strength *I* as described in: [5]

$$\kappa^{-1} = \sqrt{\frac{\epsilon_r\epsilon_0 kT}{2x10^3 N_A e^2 I}} \quad (4)$$

Where I is proportional to the number concentration of ions ψ as $I = \frac{1}{2}\sum_{i=1}^{n}\psi_i z_i^2 \propto \psi = \frac{c}{\nu}$. ν is the volume of a single ion. For $MgCl_2$; $I = \frac{5}{2}\psi = \frac{5}{2}\frac{c}{\nu}$. Rewriting equation-2 in terms of salt volume fraction, we get

$$\chi_{eff} = \chi_{ion} = \frac{\beta}{\sqrt{c}} \quad (5)$$

Where $\beta = \frac{2\pi}{3}\frac{l_B^2\sigma^2}{l^3}\sqrt{\frac{2\epsilon_r\epsilon_0 kT\nu}{10^4 N_A e^2}}$. Equation-3 now becomes,

$$\frac{F}{kT} = \frac{\phi}{N}\ln\frac{\phi}{2} + (1-\phi-c)\ln(1-\phi-c) + c\ln c + \frac{\beta}{\sqrt{c}}\phi(1-\phi-c) \tag{6}$$

Equation-6 is sufficient to describe the phase behavior of a system that phase separates at low salt concentration but undergoes mixing at higher salt concentration. To account for homotypic condensation of the polyanion, R (*i.e.,* RNA in our case), at higher salt concentration, we consider a term for the enthalpy coming from *salt-R* interactions that satisfy two conditions: (i) it must be proportional to the square of the volume fraction of R molecules $\left(\frac{\phi}{2}\right)^2$ in order to contribute to the curvature of the free energy $\frac{\partial^2 F}{\partial \phi^2}$, and (ii) it should be a function of salt concentration since our FRAP data indicate increased attraction between RNA molecules with increasing [$MgCl_2$]. These conditions lead us to consider a three body interaction term: [6]

$$\delta F_{3-body} = -\frac{\epsilon c \phi^2}{4} \tag{7}$$

Where ϵ is the strength of the interaction. This interaction may emerge from divalent cations mediating π-stacking of uracil rings in the case of poly(U) RNA [7,8]. Finally, the total free energy can be written as:

$$\frac{F}{kT} = \frac{\phi}{N}\ln\frac{\phi}{2} + c\ln c + (1-\phi-c)\ln(1-\phi-c) + \frac{\beta}{\sqrt{c}}\phi(1-\phi-c) - \frac{\epsilon c \phi^2}{4} \tag{8}$$

The stability of the mixed state is determined by the curvature of the free energy, a positive curvature indicates a locally stable homogeneous phase, while a negative curvature indicates phase separation[9]. A spinodal boundary of the system is constructed by calculating the inflection points of the free energy equation ($\frac{\partial^2 F}{\partial \phi^2} = 0$) and shown in Fig S1a. At low salt volume fraction, the free energy curvature is negative and the homogeneous phase is thermodynamically unstable. Therefore, the system spontaneously separates into *PR-rich* and *PR-poor* phases. Increasing the salt concentration causes the two-phase region to disappear and the curvature becomes positive, hence a homogeneous phase emerges. Interestingly, the curvature becomes negative again upon further increase in salt concentration, leading the system to phase separate due to contributions from the three body interaction term (Fig S1b). This is consistent with the experimentally obtained phase boundary curves of RP3-poly(U)-Mg^{2+} mixture, which is shown in Fig 2a.

In conclusion, here we presented a simple phase separation model which qualitatively captures the essential features of our ternary mixture phase behavior. Our model also predicts that for higher values of the parameter β, which is proportional to the square of linear charge density, the mixing region should vanish and the free energy curvature should remain negative throughout the range of salt concentrations (Fig S1b). This scenario can be realized for polycations containing significantly higher number of arginine residues than our present peptide system.

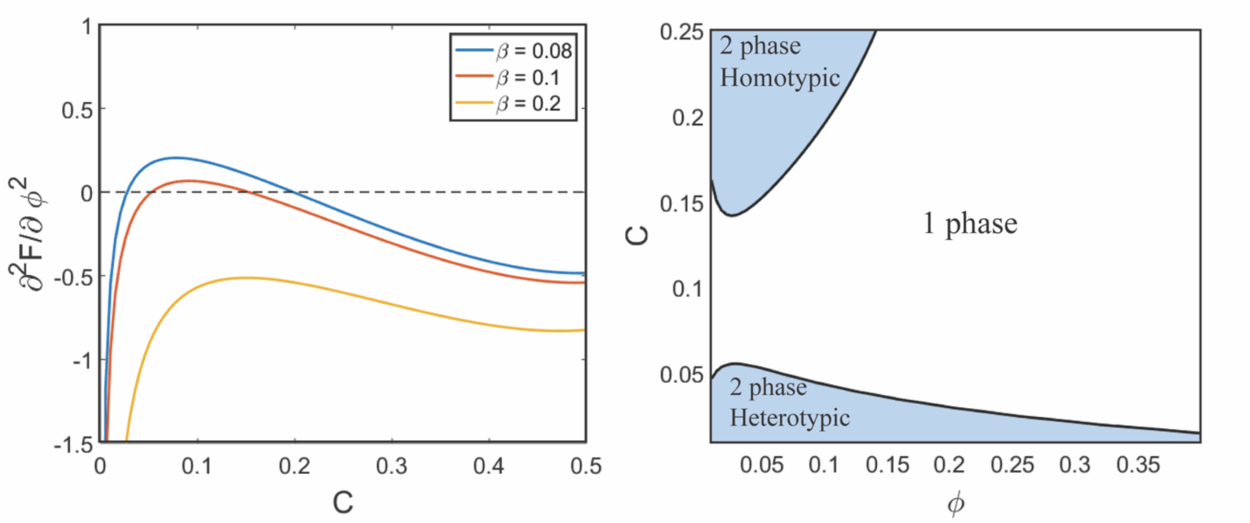

Figure S1: a. Spinodal boundary curve calculated using equation-8. Parameters used: N=1000, β = 0.1 and $\epsilon = 10$. **b.** Free energy curvature $\frac{\partial^2 F}{\partial \phi^2}$ as a function of salt concentration for the same parameters at volume fraction $\phi = 0.05$ and variable β, which is directly related to square of linear charge density.

SI note 2. Collection and Processing of Fluorescence Recovery After Photobleaching (FRAP) data.

To quantify and represent results from FRAP experiments, we collected and processed the data in the following manner.

The samples were prepared and microscopy were carried out in the manner described in the Materials and Methods. For each sample condition, we collected fluorescence intensity data for three regions of interest (ROIs, see Figure S2a for fluorescence images). These ROIs were for the bleached droplet (shown as the red square in figure S2a), a reference droplet (shown as the blue square in figure S2a), and the background intensity (shown as the green square in figure S2a). The three ROIs for each point had the same area for consistency within a single measurement. The bleached droplet was bleached using 10 iterative laser pulses of 100% laser power, while the reference droplet and background were not bleached. The sample was then imaged to follow recovery until the bleached droplet returned to the fluorescence intensity of the reference droplet (Fig S2a-b). This comparison to the reference droplet was essential in that it allowed us to correct for any issues in general photobleaching of the sample. The background was taken to ensure a low, stable baseline. Figure S2b shows the fluorescence intensity points (averaged over the ROI) taken for the sample in figure S2a.

For each point, we corrected for photobleaching effects using the equation:

$$I_N = \left(\frac{I_B}{I_{B0}}\right)\left(\frac{I_{R0}}{I_R}\right)$$

I_N refers to the normalized intensity. I_B and I_R refer to the fluorescence intensity of the bleached droplet and the reference droplet, respectively. I_{B0} and I_{R0} refer to the fluorescence intensity of the bleached droplet and the reference droplet at time point 0, prior to bleaching. The term $\left(\frac{I_B}{I_{B0}}\right)$ in the equation normalizes the intensity of the bleached droplet to the initial intensity, while the term $\left(\frac{I_{R0}}{I_R}\right)$ (for the reference droplet) corrects for any global photobleaching effects (due to prolonged imaging during recovery) beyond the effects of the specific FRAP photobleaching and fluorescence recovery. The normalized intensity data are shown in figure S2c (black dots). These data were fitted with an exponential equation:

$$y = y_0 + A_1 e^{x/\tau}$$

We obtained the time constant τ using this fit (shown as the red line in figure S2c), which gave us a measure of the recovery of the bleached droplet. To correct this value for the ROI differences between points, we scaled the τ value using the area of the original ROI of the measurement $\left(\frac{\tau}{Area(ROI)}\right)$. This whole process was repeated for several droplets (3-6) bleached at

every condition. The mean scaled FRAP time was taken at each point with error bars representing one standard deviation. All FRAP data were processed and represented using this procedure throughout the study.

See next page for Figure S2. Image data for the different conditions are available upon request.

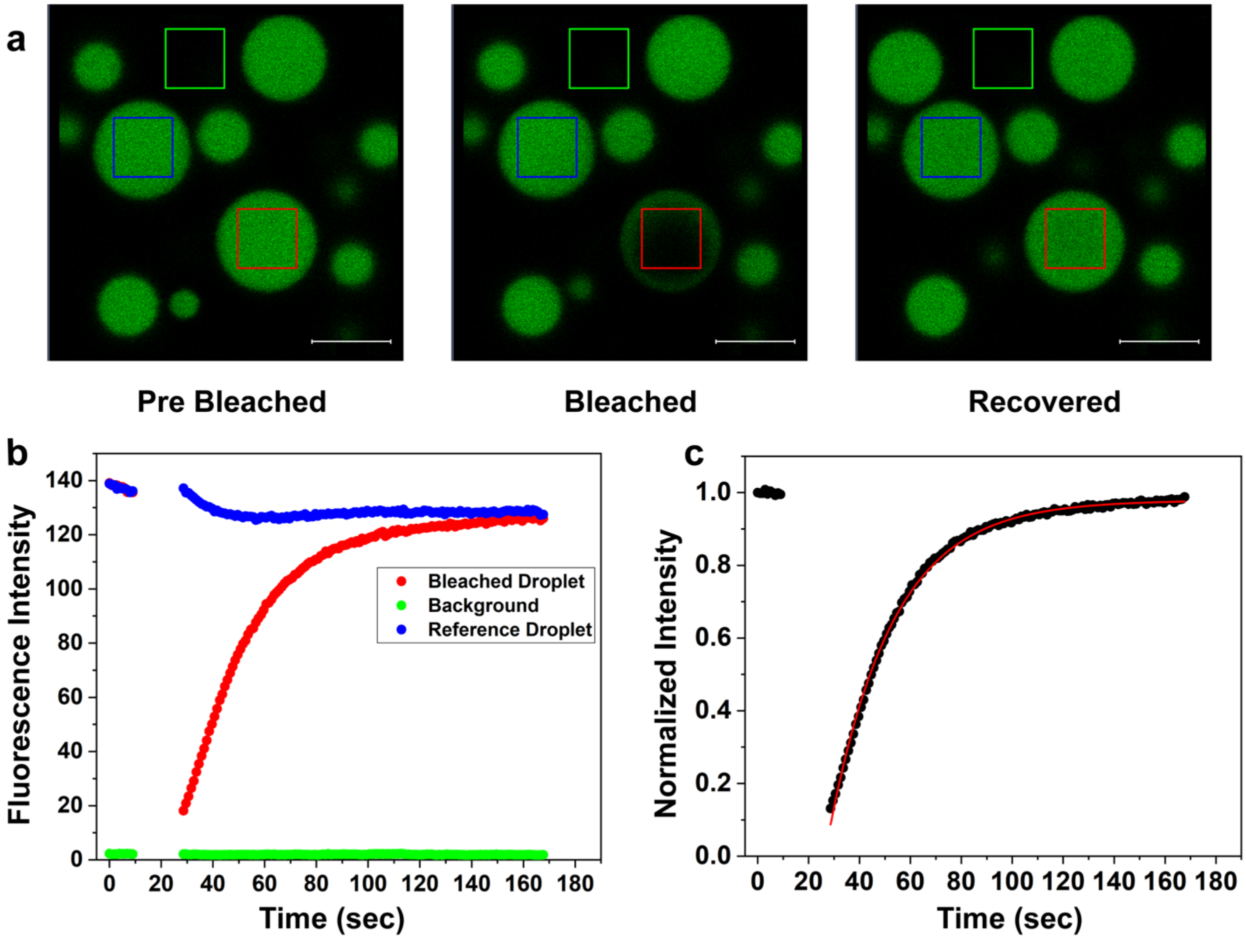

Figure S2: a. Confocal fluorescence microscopy images showing FRAP of polyU coacervates with the bleached droplet (red square), the reference droplet (blue square), and the background (green square). Scale bars represent 5μm. ROI Areas = 13.94μm^2. **b.** Raw fluorescence intensity curves from the ROIs depicted in the fluorescence microscopy images. **c.** Normalized intensity data (black dots) obtained as discussed in the text above. The red line depicts the exponential fit. All data in this figure were taken using the condition (2mg/mL polyU, 600mM $MgCl_2$, 10% PEG, 0.5μM FAM-UGAAGGAC), represented in one of the points in figure 4b.

Supplementary Figure 3

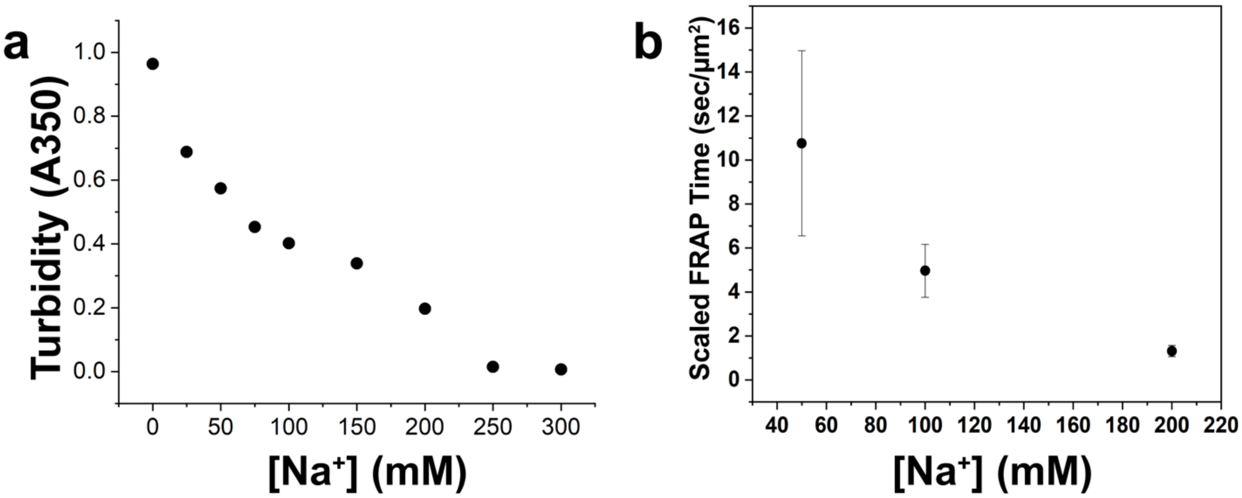

Figure S3: Sodium concentration leads to decreased formation and variation of RP3-polyU droplet fluidity. a. Solution turbidity measurements of RP3-polyU droplets as a function of [NaCl]. ([RP3] = 500μM, 0.6x polyU wt/wt) **b.** Scaled FRAP in RP3-polyU droplets as a function of [NaCl]. ([RP3] = 500μM, 0.6x polyU wt/wt)

Supplementary Figure 4

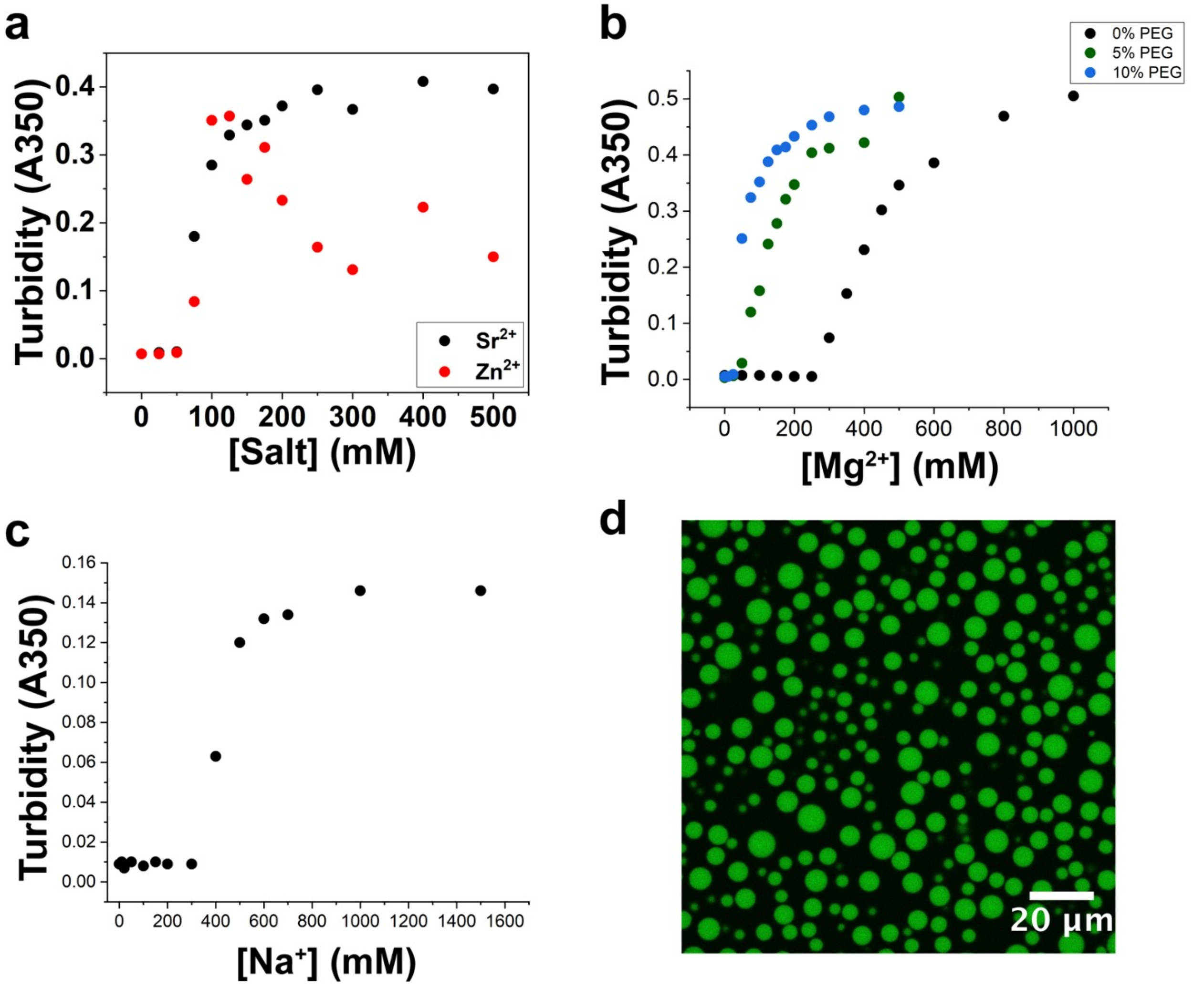

Figure S4: PEG influences the phase boundaries in the presence of divalent and monovalent salts. a. Solution turbidity measurements of polyU droplets as a function of [$SrCl_2$] and [$ZnCl_2$]. (1mg/mL polyU) **b.** Solution turbidity measurements of polyU droplets as a function of [$MgCl_2$] in the presence of PEG. (1mg/mL polyU) **c.** Solution turbidity measurements as a function of [NaCl]. (1.5 mg/mL polyU, 10% PEG) **d.** Confocal fluorescence image showing polyU droplets in the presence of NaCl. (2mg/mL polyU, 1M NaCl, 10% PEG)

Supplementary Figure 5

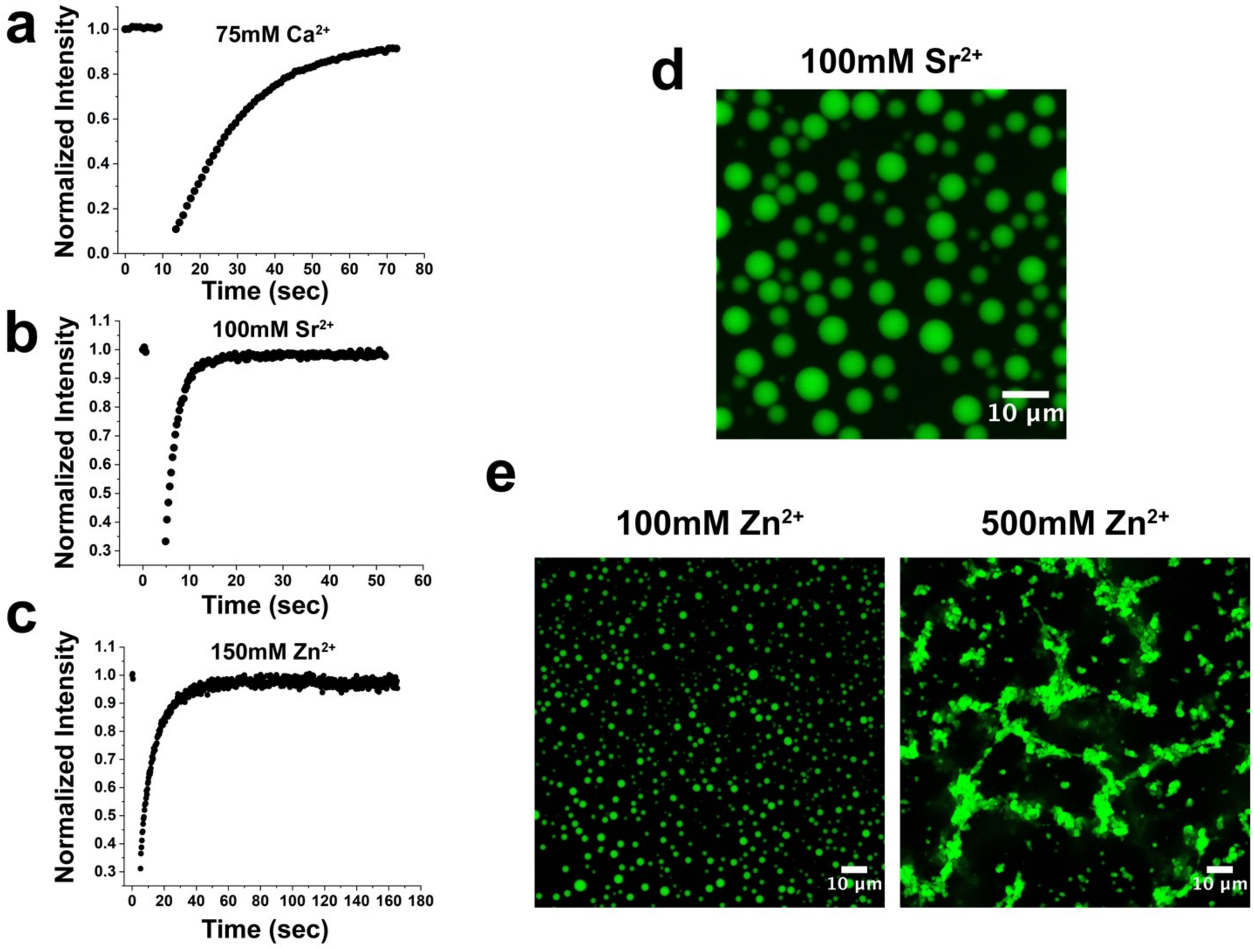

Figure S5: Multiple divalent cations drive formation of fluid homotypic polyU droplets. a. FRAP curve of homotypic polyU droplets showing fluidity and full recovery. (75mM $CaCl_2$, 2mg/mL polyU) **b.** FRAP curve of homotypic polyU droplets showing fluidity and full recovery. (100mM $SrCl_2$, 1mg/mL polyU) **c.** FRAP curve of homotypic polyU droplets showing fluidity and full recovery. (150mM $ZnCl_2$, 1mg/mL polyU) **d.** Confocal fluorescence image showing polyU droplets in the presence of $SrCl_2$. (1mg/mL polyU, 100mM $SrCl_2$) **e.** Confocal fluorescence images showing polyU droplets in the presence of 100 mM $ZnCl_2$ (left panel) and polyU aggregates in the presence of 500mM $ZnCl_2$ (right panel). (1mg/mL polyU in both panels)

Supplementary Figure 6

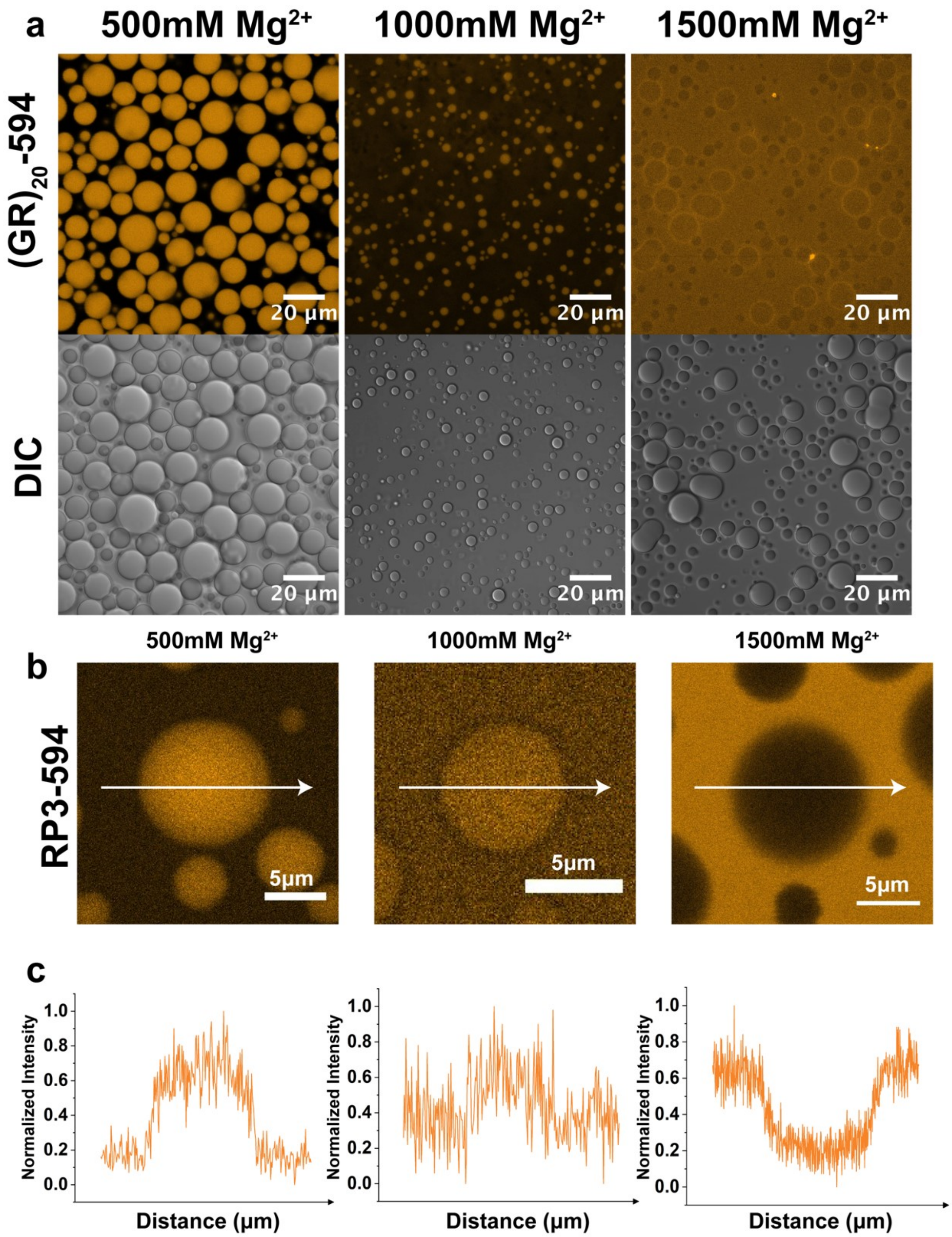

Figure S6: PolyU droplets exclude positively-charged peptides at high Mg^{2+} concentration. **a.** Confocal fluorescence and DIC images of polyU droplets with Alexa594-labeled GR20 as a fluorescent probe. ([polyU] = 1.5mg/mL, 0.5µM GR20-AF594). **b.** Confocal fluorescence images showing Alexa594-labeled RP3 partitioning into homotypic polyU droplets. ([polyU] = 1.5mg/mL, 0.5µM RP3-AF594) **c.** Fluorescence intensity profiles of the images in b., with the distance axis corresponding to the white arrows.

Supplementary Figure 7

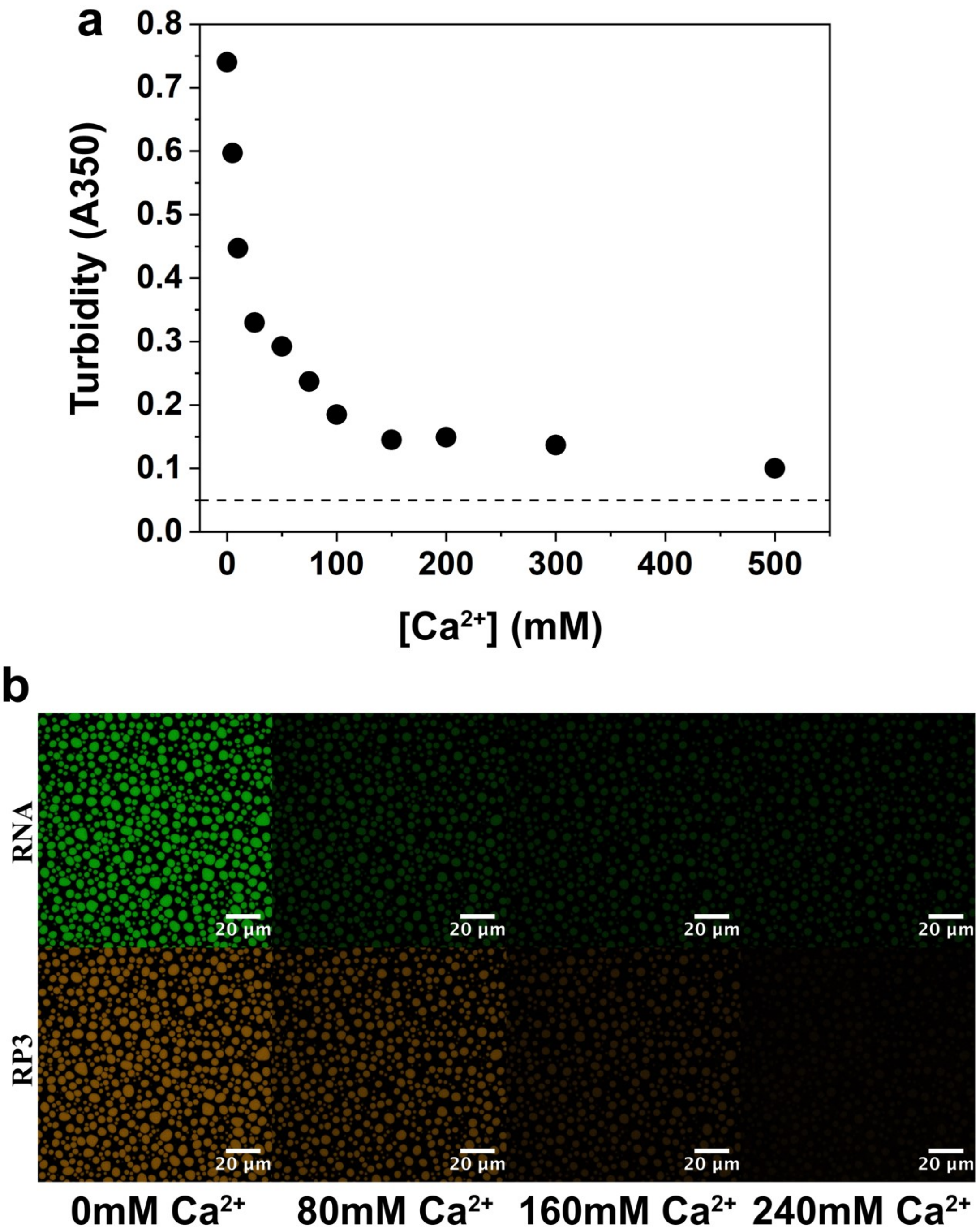

Figure S7: Heterotypic and homotypic droplets can overlap, showing no window of miscibility. a. Solution turbidity measurements of RP3-polyU droplets as a function of [$CaCl_2$]. The dotted line represents our established turbidity cutoff for phase separation at A_{350} = 0.05. ([RP3] = 500μM, 0.6x polyU wt/wt) **b.** Confocal fluorescence microscopy images of droplets in a sequential titration of $CaCl_2$. Predicted concentration of Ca^{2+} is shown below the frames. ([RP3] = 500μM, 0.6x polyU wt/wt, 0.5μM FAM-UGAAGGAC, 0.5μM RP3-AF594)

Supplementary Figure 8

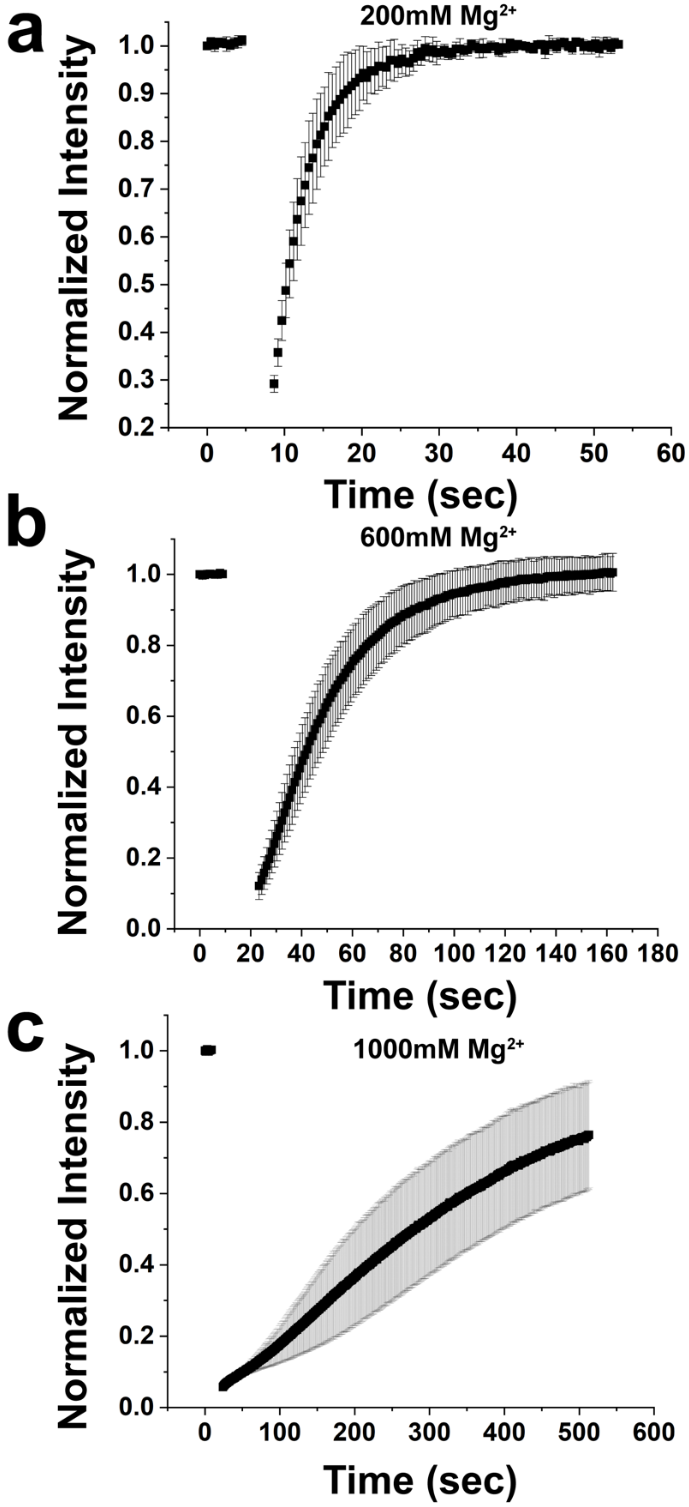

Figure S8: Fluorescence recovery after photobleaching at varying Mg^{2+} concentrations. a. 200mM Mg^{2+} ([polyU] = 2 mg/ml, 10% PEG) **b.** 600mM Mg^{2+} ([polyU] = 2 mg/ml, 10% PEG) **c.** 1000mM Mg^{2+} ([polyU] = 2 mg/ml, 10% PEG)

Supplementary Figure 9

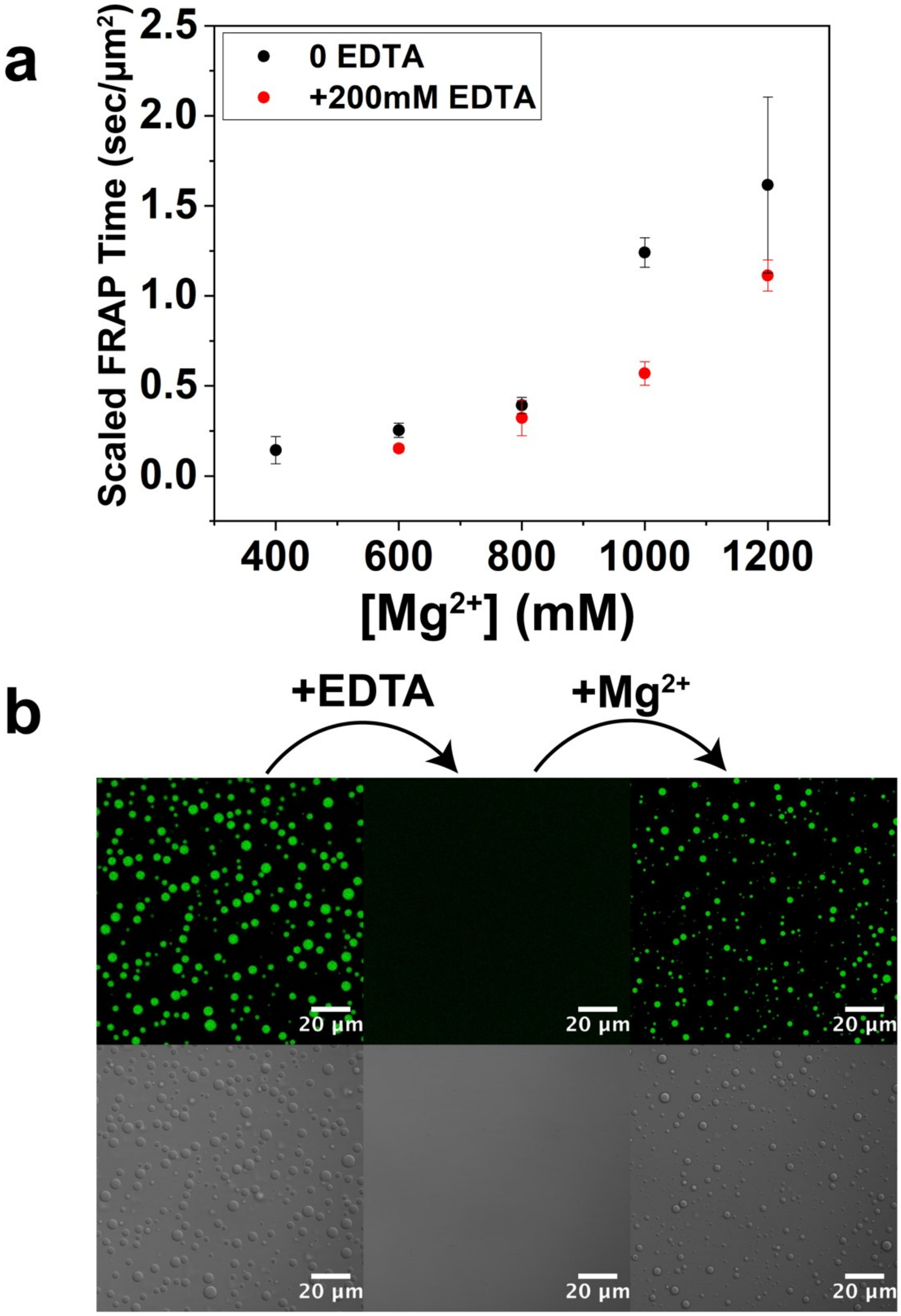

Figure S9: EDTA reverses the effect of Mg^{2+} on homotypic polyU droplets. a. Scaled FRAP times in the absence and presence of EDTA. (2mg/mL polyU) **b.** Fluorescence and DIC microscopy images showing reversible dissolution and formation of polyU droplets by consecutive addition of EDTA and $MgCl_2$. (Frame1: 1.5mg/mL polyU, 35mM $MgCl_2$, 10% PEG, 0.5μM FAM-UGAAGGAC - Frame 2: Addition of 50mM EDTA - Frame 3: Addition of 100mM $MgCl_2$)

Supplementary Figure 10

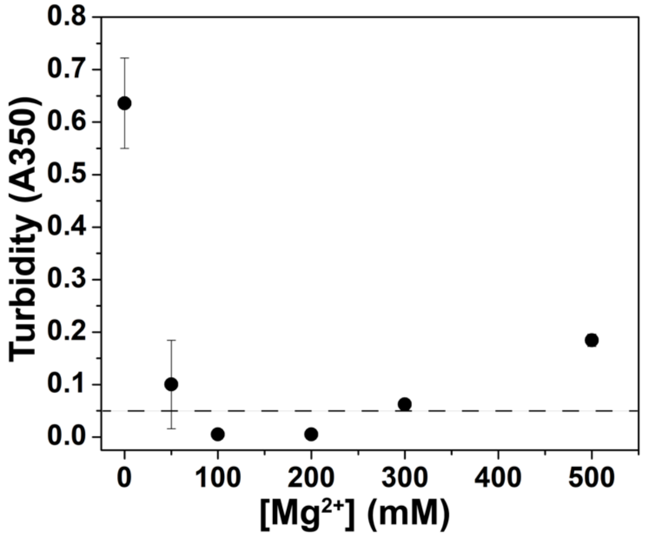

Figure S10: RNA coacervates undergo a switch like transition with a lysine-based peptide. Solution turbidity measurements of a sequential $MgCl_2$ titration showing droplet switching. The dotted line represents our established turbidity cutoff for phase separation at $A_{350} = 0.05$. ([KGKGKGKGKGKGKGK] = 500μM, 1.5x polyU wt/wt)

Estimated Partition Coefficients

Table S1

	10mM Mg^{2+}, 500μM RP3, .6X polyU	100mM Mg^{2+}, 500μM RP3, .6X polyU	400mM Mg^{2+}, 1mg/mL polyU	1000mM Mg^{2+}, 1mg/mL polyU
[6FAM]UGAAGGAC	**2438 ± 422**	**142 ± 10**	**57 ± 3.7**	**91 ± 4.3**

Table S2

	10mM Mg^{2+}, 500μM RP3, .6X polyU	500mM Mg^{2+}, 1mg/mL polyU	1000mM Mg^{2+}, 1mg/mL polyU	1500mM Mg^{2+}, 1mg/mL polyU
RP3-AF594	**11386 ± 1887**	**2.9 ± 0.1**	**1.4 ± 0.04**	**0.77 ± 0.03**
GR20-AF594	**5721 ± 767**	**65 ± 13**	**3.8 ± 0.46**	**0.95 ± 0.03**

Table S3

	10mM Mg^{2+}, 500μM RP3, .6X polyU	500mM Mg^{2+}, 1mg/mL polyU
[6FAM]U10	**759 ± 144**	**2.3 ± 0.1**
[6FAM]A10	**N/A**	**1134 ± 95**
AF488	**39 ± 2.3**	**1.3 ± 0.04**
eGFP-MBP-AF488	**0.39 ± 0.015**	**0.095 ± 0.0088**
α-synuclein-AF488	**4.4 ± 0.17**	**0.13 ± 0.0057**
Hsp27-AF594	**14 ± 2.1**	**0.72 ± 0.083**

These tables represent the apparent partitioning estimated from fluorescence intensity measurements of droplets and the background, as described in the Microscopy section of the Materials and Methods. This same procedure is widely used in the field. The error (1 standard deviation) was determined based on several intensity measurements for each sample. While additional considerations can apply to obtaining absolute partition coefficients (see Materials and Methods), the numbers presented here provide a reliable estimate within the order of magnitude for preferential partitioning vs. exclusion, which were used for our conclusions in the paper.

Table S4 – Proteins/Peptides Molecular Weight and Theoretical PI

	**Molecular Weight (kDa)**	**Theoretical pI**
eGFP-MBP	77.6	5.03
α-synuclein	14.5	4.67
Hsp27	22.8	5.98
RP3	1.9	12.3
$(GR)_{20}$	4.4	12.95

References

1. Flory, P. J. Thermodynamics of High Polymer Solutions | Browse - Journal of Chemical Physics. *J. Chem. Phys.* **660,** 13–15 (1941).

2. Overbeek, J. T. G. & Voorn, M. J. Phase separation in polyelectrolyte solutions. Theory of complex coacervation. *J. Cell. Comp. Physiol.* (1957). doi:10.1002/jcp.1030490404

3. Lyklema, J. *Fundamentals of interface and colloid science: soft colloids*. (Academic Press, 2005).

4. Spruijt, E., Westphal, A. H., Borst, J. W., Cohen Stuart, M. A. & Van Der Gucht, J. Binodal compositions of polyelectrolyte complexes. *Macromolecules* **43,** 6476–6484 (2010).

5. Robins, M. & Fillery-Travis, A. Colloidal dispersions. Edited by W. B. Russel, D. A. Saville & W. R. Schowalter, Cambridge University Press, Cambridge, UK, 1989, xvii + 506 pp., price: £60.00. ISBN 0 521 34188 4. *J. Chem. Technol. Biotechnol.* **54,** 201–202 (2010).

6. Wei, M. T. *et al.* Phase behaviour of disordered proteins underlying low density and high permeability of liquid organelles. *Nat. Chem.* **9,** (2017).

7. McFail-Isom, L., Shui, X. & Williams, L. D. Divalent cations stabilize unstacked conformations of DNA and RNA by interacting with base II systems. *Biochemistry* **37,** 17105–17111 (1998).

8. Gallivan, J. P. & Dougherty, D. A. A computational study of cation-π interactions vs salt bridges in aqueous media: Implications for protein engineering. *J. Am. Chem. Soc.* **122,** 870–874 (2000).

9. Rubinstein, M. & Colby, R. H. Polymer physics. *Polymer International* 440 (2003). doi:10.1002/pi.1472
